# Asthma Case Cluster during Renovation of a Water-Damaged and Toxic Building

**DOI:** 10.3390/microorganisms7120642

**Published:** 2019-12-03

**Authors:** Saija Hyvönen, Hannu Syrjala

**Affiliations:** 1 Pihlajalinna, 90140 Oulu, Finland; saija.hyvonen@pihlajalinna.fi; 2 Departments of Infection Control and Process Development, Future Hospital OYS 2030 program, Oulu University Hospital, Box 21, 90029 OYS, Finland

**Keywords:** renovation, occupational asthma, indoor dust toxicity, sperm motility, fungi, bacteria

## Abstract

Background: An association between fungal exposure at work and asthma onset has been shown, but a causal relationship between them has not beTanle en established. Methods: The study describes an asthma cluster in workers in a building under renovation. Before renovation the work site had significant water damage, technical deficiencies, and ventilation problems. Worker protection was insufficient during renovation. In the building, toxicity was determined from dust as well as from cultured dust. Toxicity analysis was conducted in vitro using the boar spermatozoa motility assay. Results: During the 8-month renovation period, among 290 workers, 21 (7.2%) experienced new-onset asthma (9 women, 42.9%; 12 men, 57.1%; median age, 43 years (range, 30–60 years)). At the renovation site, they had been exposed to areas where remarkable toxicity was demonstrated in vitro. One year later, 13 (61.9%) of them still had moderate disease, and three (14.8%) had severe disease. Most patients had a poor response to inhaled corticosteroids. Conclusions: This study documents a clear temporal association between occupational exposure during renovation of a water-damaged building and a cluster of 21 new occupational asthma cases. In addition, dust and cultured dust from their work spaces showed remarkable toxicity based on inhibition of boar sperm motility in vitro.

## 1. Introduction

Asthma is a chronic respiratory disease affecting hundreds of millions people worldwide [1]. It is heterogeneous, and respiratory symptoms and airflow obstruction vary. Different categories of asthma have been identified based on clinical and physiological features [2,3,4]. From the point of immunopathology, for example, asthma can be divided into eosinophilic, non-eosinophilic, and mixed granulocytic disease [5].

Eosinophilic asthma includes most asthma that arises during childhood and about 50% of asthma patients in adulthood. This eosinophilic inflammation is regulated by the T helper 2 (T2) cytokines interleukin (IL)-4, IL-13, and IL-5. In this high type 2 immnunity asthma, patients show a good response to inhaled corticosteroid (ICS) treatment [1]. In low type 2 immunity asthma with increased concentrations of the T1 cytokines IL-17 and IL-23, neutrophils predominate in bronchial specimens [1]. This late-onset asthma is usually associated with a poor response to ICS treatment [6].

About 5–20% of adult-onset asthma cases are work-related occupational asthma (OA) [7,8]. More than 300 causative agents in OA have been identified and can be classified into high-molecular-weight (≥ 5000 g/mol) proteins of vegetal or animal origin, or low-molecular-weight (LMW) chemicals [9,10]. OA eosinophilic inflammation is a feature of asthma caused by high-molecular-weight proteins, but in LMW OA, which involves a poor steroid response, the neutrophilic inflammatory response is crucial [11].

According to a recent literature review of indoor mold exposure and asthma [12], longitudinal prospective studies published between 2006 and 2017 show evidence of a link between indoor mold exposure at work and asthma, but without establishing a causal relationship. Here we describe a cluster of 21 new asthma cases involving a poor ICS treatment response. The patients acquired the disease during an 8-month renovation of a dampness-damaged building.

A prior study using an in vitro boar sperm motility assay showed that dust samples from buildings with reported adverse health effects were toxic [13]. Furthermore, we have previously shown, using a similar assay, an association between common building-related symptoms in teachers’ working environments and toxic dust and airborne microbes [14]. In this study, we were interested in determining whether any corresponding toxicity to boar sperm could be demonstrated in the dust at the work site of these 21 asthma patients.

## 2. Material and Methods

### 2.1. Background and Symptoms of Workers

The number of office workers in the building undergoing the renovation in Oulu in Northern Finland increased from 190 in 1988 to 290 by 2016. Since 2004, these workers have increasingly experienced symptoms such as irritation of the nose, eyes, and skin, vocal cord problems, cough, urticaria, and cardiac and neurologic symptoms. Between 2004 and 2015, several improvements were made in the building’s structures and ventilation without sufficient improvement of building-related symptoms. In interviews, workers revealed that the building had had several sewer pipe overflows over the years on the lowest floors, which were not appropriately repaired.

During autumn 2016, a large reconstruction project was started. The work was proceeding as a typical renovation without sufficient protection of areas under reconstruction, and normal office work continued in those areas. The office workers described the air in their workspace as being filled with dust. During that time, 138 of 290 workers (47.6%) contacted their occupational health care unit because of lower airway tract symptoms, upper airway tract irritation, infections, and eye and skin symptoms. In February 2017, city authorities terminated the renovation because of these health issues.

According to the secretary of the Ethical Committee of Oulu University Hospital, approval for this study from the committee was unnecessary because the study is a register-based survey. Moreover, exemption from consent was obtained because the data retrieved from databases had already been recorded for patient treatment purposes.

### 2.2. The Workplace

The 9000-m^2^ office building was built in 1988, consists of five floors, and is located in Finland. The main structures are a slab-on-grade concrete floor, thin-cell concrete walls with a clinker tile surface, and a flat roof with an inside steered sewerage drainage system. There is a forced-air ventilation system in the building.

There have been several episodes of water damage over the decades. Such damage had been observed in the thin-cell concrete walls near the windows, where it was due to faults in the seams, and it is likely that similar damage was present behind many other seams. In addition, air leaks were present near the windows and in the inner wall panels, opening the way to airborne microbial impurities from outside. In some office rooms, this problem manifested as an odor of mold. In addition to the problems with the outside walls, the occupants also reported significant leaks in the roof drainage pipes, resulting from a failure in the rainwater control system.

The air pressure in the building was highly negative because of the ventilation system. Negative pressure increases air leaks through structures and can worsen indoor air quality if the air flows through microbe-contaminated materials. In addition, the negative air pressure led to make-up air being drawn from sewer pipes, bringing sewage odor into the building. Ultimately, the number of occupants had surpassed the original plan by 50%, exceeding the ventilation system’s designed capacity. In addition, industrial mineral fiber had been detected, and there were multiple reports of dirt and dust. No other known respiratory disease-causing agents such as organic dust, animal allergens, or industrial chemicals were reported in the building.

A thorough renovation was started in 2016 in the lower floors. The renovation work produced a large amount of dust. Protection in this case was insufficient, and the dust was spread all over the building via technical chases and inlets. Dust layers were seen on surfaces on even the fifth floor. The dust contained potentially harmful microbes from the renovation process, and most of the occupants were exposed to it.

Several water-damage indicator microbes were found in cultured samples from surfaces in different floors of the building. The following molds were identified: *Aspergillus section Versicolores*, *Aspergillus section Restricti*, *Aureobasidium*, and species of *Engyodontium*, *Eurotium*, *Fusarium*, *Phoma* and *Paecilomyces*, *Sphaeropsidales*, and *Wallemia*. The bacterial genus *Actinobacteria* was also identified.

### 2.3. Toxicity Measurements of Workplace Samples

The specimens for toxicity analyses were obtained from the rooms and spaces where the asthma patients had worked daily. These specimens were obtained after these asthma patients had been transferred to other workplaces. Briefly, the dust was collected by wiping the horizontal surfaces >1 m above the floor level with sterile microfiber cloth. Dust samples were divided in three parts. The first part was extracted in ethanol, evaporated to dryness, and re-dissolved in ethanol to a concentration of 10 mg/mL [13]. The other parts of the dust were cultured in malt extract (70,167, Sigma-Aldrich, Merck KGaA, Darmstadt, Germany) agar (05039, Sigma-Aldrich, Merck KGaA, Darmstadt, Germany) and tryptic soy agar (22,091, Sigma-Aldrich Merck KGaA, Darmstadt, Germany) plates for fungi and bacteria, respectively, for 4 weeks. The microbe biomass was collected, extracted in ethanol, evaporated to dryness, and re-dissolved in ethanol to a concentration of 10 mg/mL [13]. The method used for handling the samples has been described in detail previously [13,14].

Dust samples were analyzed for toxicity of three kinds: Dust toxicity, fungal toxicity from cultured dust, and bacterial toxicity from cultured dust. The toxicity analysis assay was based on inhibition of boar spermatozoa motility. The exposed spermatozoa were assessed by microscopy for motility until identification of the lowest exposure concentration causing >50% of the spermatozoa to lose motility relative to vehicle only (ethanol). The half maximal effect concentration or EC_50_ was expressed in µg/mL and a lower EC_50_ indicates higher toxicity. The method used for toxicity analysis has been described in detail previously [13,14].

## 3. Results

### 3.1. Participants

Over the course of 8 months, between January and August in 2017, 21 new asthma cases were diagnosed (Table 1), involving nine women (42.9%) and twelve men (57.1%). The diagnosis was made in their occupational health care unit in 17 cases by two occupational health specialists. One of them is the first author (S.H.). Four histamine provocation test-positive cases were diagnosed at Oulu University Hospital. The median age of the 21 asthma patients, who comprised 7.2% of the 290 workers, was 43 years (range, 30–60 years). Their median body mass index was 27 (range, 22–33), with five (23.8%) having a value > 30. Two patients were ex-smokers, one had a history of atopy and allergic rhinitis, and two more had allergic rhinitis (Table 1). Three (14.3%) patients reported asthma in their parents or siblings. Before their asthma diagnoses were verified, 18 had a cough (85.7%), and 16 had shortness of breath (76.2%).

The diagnostic criteria for their asthma [15] and their asthma medication needs at 1 year following diagnosis are presented in Table 1. The severity of their asthma was as follows [16]: Mild in four (19.0%; one step 1, three step 2); moderate in 13 (61.9%; step 3); and severe in three (14.8%; step 4). The duration of working in the building before the renovation and asthma diagnosis varied from 3 months to 28 years, with a median time of 6 years. In four cases, the duration of working history was not available. The prevalence of asthma in this workplace increased from 10% in 2016 to 17.2% in 2017.

According to follow-up one year later in 2018, most of the diagnosed patients had continuing symptoms of asthma and needed to use several asthma medications (Table 1). All of them had been transferred to other buildings. Two of the workers did not have continuous medication after the transfer, but one of the two reported needing occasional medication because of lower airway tract symptoms in some circumstances (step 1). The other of the two patients (patient 13) had poor adherence and did not use asthma medication despite having symptoms (Table 1). Five of the workers have suffered from symptoms significantly enough to need sick leave for 4–8 weeks. Four more needed part-time sick leave to cope, and another four had applied for part-time pensions after the asthma diagnosis.

### 3.2. Toxicity Measurements in the Workplace

Table 2 shows the results of boar-sperm toxicity analyses of dust and cultured dust. Each of the 21 asthma patients had worked in the area where strong toxicity of at least one kind was demonstrated. Strong dust toxicity (EC_50_ = 6.5–12.5 µg/mL) was observed in 14 cases (66.6%), four with very strong toxicity (EC_50_ < 6 µg/mL). In cultured dusts, strong mold toxicity (EC_50_ ≤ 16 µg/mL) was observed in five cases (23.8%), and strong bacterial toxicity (EC_50_ ≤ 9 µg/mL) in 20 cases (95.2%). The following strong toxicity combinations were observed: all three methods in five cases (23.8%); toxicity of dust together with cultured dust mold in five cases (23.8%), and together with cultured bacterial dust toxicity in 14 cases (66.7%); and in cultured dust, both mold and bacterial toxicity in seven cases (33.3%).

## 4. Discussion

Our results show for the first time a temporal association between a building renovation for dampness-related damage and a cluster of new OA cases in a workplace. In addition, dust and cultured dust from the work spaces of these OA patients showed remarkable toxicity based on inhibition of boar sperm motility in an in vitro assay.

In recent years, understanding of the pathogenesis of asthma has expanded in terms of airway inflammation, airway hyper responsiveness, and airway remodeling [5]. For example, both low type 2 and high type 2 inflammatory conditions augment hyperresponsiveness. However, the complexity of inflammatory processes causing airway changes in asthma remains a challenge [17,18]. LMW toluene diisocyanate (TDI; 174.2 g/mol) is one of the most common causes of OA worldwide. Studies on the pathogenesis of TDI OA have shown that both eosinophilic (high type 2 airway inflammation) and non-eosinophilic (low type 2 airway inflammation) mechanisms are important [11]. The pathogenesis of TDI-induced asthma in at least one mouse model depends on the Th17 response: IL-17A suppresses TH2 inflammation with eosinophil recruitment, whereas IL17F drives Th17 inflammation and neutrophil increase in airways [19].

Although we lacked any data for some of the clinical and physiological variables usually used for classification of asthma clusters, our asthma patients with a poor ICS response, male predominance, and a median age of 43 years showed an unlikely grouping into asthma clusters 1 and 2. Moreover, only three patients reported asthma in their parents or siblings, one had atopy, and three suffered from allergic rhinitis. Asthma associated with clusters 3 and 4 involves higher chitinase-like protein YKL-40 serum concentrations than does the disease in clusters 1 and 2 [20]. YKL-40 is associated with airway inflammation and causes smooth muscle proliferation, airway remodeling, and bronchial obstruction. According to airway transcriptome analyses, patients whose asthma can be categorized into clusters 3 and 4 have an activation of non-T2 inflammatory pathways [20].

Lung microbiome studies with 16S ribosomal sequencing have shown that healthy humans also harbor many uncultured microbes in the respiratory airways, although the number and spectra of microbes vary in eosinophilic and neutrophilic asthma [21,22,23,24]. The respiratory tract in asthma shows enrichment of Gammaproteobacteria such as *Haemophilus* and *Moraxcella*. In one mouse model, *Haemophilus influenzae* infection propagated an augmented neutrophilic inflammation with an increase in the IL-17 response [25]. In healthy persons, like our workers before the renovation work began, the prevailing lung inflammation process is associated with a Th17 phenotype [26].

In a large European OA study, the median duration of exposure to high-molecular-weight asthma-associated agents before disease onset was 96 months (8 years), and it was 61 months (5 years) with LMW agents [11]. In our series, the median working history in the building (6 years) was in agreement with these exposure durations. The shortest working time in the building before the onset of asthma was only 3 months, and all cases occurred within 8 months of the start of the renovation, suggesting acutely hazardous conditions. We could identify no other explanation for this asthma cluster. For example, no known organic dust or animals or industrial chemicals were identifiable in the building.

When toxigenic fungi grow on wallpaper, aerosolisation of non-volatile mycotoxins may occur even during normal ventilation conditions [27]. In outdoor air, fungal spores can be detected throughout the year at very high counts, while more than 50,000 fungal spores/m^3^ of air per day can be observed from June to August in Leicester, UK [28]. With these high outdoor counts, it is rational to infer that spore counts and even secondary metabolites of toxic microbes may increase many fold in dusty indoor air during a renovation.

We do not have an exact answer as to how these workers acquired asthma in this building. There are, however, several possible explanations. The number of workers had surpassed the original plan by 50%, and the ventilation system was not designed for so many. Furthermore, there had been several episodes of water damage over the decades and water-damage indicator microbes were found in cultured samples from surfaces on different floors of the building. The air pressure in the building was highly negative because the ventilation system allowed increased air leakage through building structures. During the renovation the protection of workers was insufficient and they described the air in their work space as being filled with dust. The dust and cultures from dust were markedly toxic in an in vitro assay. All the above-mentioned factors together constituted a harmful environment, where repeated and long-lasting exposure—some workers had 12-h shifts—likely contributed to chronic damage.

Our OA patients had a poor steroid response suggesting the possibility of low type 2 immunity asthma, which has been reported to have increased concentrations of the T1 cytokines IL-17 and IL-23, neutrophils predominate in bronchial specimens [1]. It has been shown earlier also that in LMW OA with a poor steroid response, the neutrophilic inflammatory response is crucial [11]. It is interesting to note that all mycotoxins are LMW secondary metabolites of filamentous fungi [29]. Also valinomycin, a bacterial toxin of *Actinobacteria*, is a LMW compound [30]. So, in theory the possibility for the exposure to these LMWs was a real option in the environment of these OA patients.

An in vitro assay based on inhibition of boar sperm motility has been shown to be a sensitive tool for tracking bacterial and fungal toxins like valinomycin, cereulide, amylosin, stephacidin A and B, and several peptaibols associated with human indoor illness [13,31,32,33,34,35,36,37]. We have earlier seen that building-related symptoms were significantly more common among teachers who worked at least 7 h/week in classrooms where dust samples were highly toxic to sperm (impairing the motility of boar sperm cells), even if no obvious toxicity was observed in the classroom [14]. Teacher symptoms under these conditions appeared to be reversible and were alleviated during vacations. The situation was different in these OA patients. Most of the 21 asthma patients were in good health before the renovation process but showed a dramatic physical decline because of asthma. OA implies long-lasting consequences. One year after their diagnoses, most still had moderate (62%) or severe (15%) diseases [16], and 2 years later 20 of 21 of these occupational asthma patients needed still asthma medication in 2019.

Our study has some limitations. The large panel of clinical and physiological variables usually used for the characterization of asthma clusters was not available because of ambulatory treatment in the occupational health care unit. The diagnosis of OA should be confirmed by specific inhalation challenge (SIC) whenever possible [38]. In our cases, with a severely damaged and toxic building, it would have been difficult to perform SIC with certain agents. Moreover, our patients experienced severe symptoms if they needed to visit their office for some reason, and SIC thus was considered an unethical approach. As secondary prevention of OA, the most important factor is avoiding the causative exposure [38]. Our patients were unable to do so because the association between renovation and these new asthma cases was considered unlikely. The microbes causing bacterial and fungal toxicity in dust cultures were not identified, which is also a limitation in our study. Furthermore, because of the nature of our study, a description of a cluster of patients with OA, no a priori hypothesis was not able to test, but there were several elements with linked together these OA cases and the harmful environment, where these 21 patients had worked.

## 5. Conclusions

In conclusion, to our knowledge, we describe the first cluster of patients with OA temporally linked to a building renovation. Furthermore, we found a remarkable toxicity of dust and cultured dust samples from this workplace as defined by inhibition of boar sperm motility in an in vitro assay.

## Figures and Tables

**Table 1:** Clinical data for 21 patients who acquired occupational asthma during an 8-month renovation of their building.

No.	Sex	Smoking	Atopy	Allergic Rhinitis	FVC	FEV1	Diagnostic Criteria [15]	Asthma Medication	**Severity** [16]
1	Male	no	no	no	4.98l/92%	4.10l/89%	2	ICS, LABA, salbutamol daily	G3
2	Male	no	no	no	5.53l/102%	4.32l/99%	2	ICS, LABA, montelukast, salbutamol daily	G3
3	Female	no	no	yes	4.24l/94%	3.12l/84%	3	ICS, LABA, salbutamol	G3
4	Female	no	no	no	4.17l/86%	3.54l/88%	2	ICS and LABA re-started in spring 2018	G3
5	Female	no	no	no	2.87l/87%	1.34l/51%	1	ICS, LABA, montelukast, salbutamol weekly	G3
6	Female	yes	N.A.	no	2.51l/75%	1.48l/54%	1	ICS, LABA, tiotropium, salbutamol weekly	G4
7	Female	no	no	no	6.36l/147%	5.00l/135%	2	ICS, LABA, salbutamol during exercise	G3
8	Male	no	no	no	4.34l/82%	3.78l/87%	2	ICS, LABA, salbutamol during exercise	G3
9	Male	no	no	no	4.45l/93%	3.00l/75%	2	salbutamol occasionally	G1
10	Male	no	no	no	3.79l/79%	2.96l/77%	1	ICS, LABA, salbutamol weekly	G3
11	Male	no	no	no	5.73l/96%	4.40l/94%	2	ICS, LABA, montelukast	G3
12	Male	no	no	no	4.02l/76%	2.93l/71%	1,2	ICS, LABA, montelukast	G3
13	Male	no	no	N.A.	2.59l/51%	2.10l/51%	1	ICS, LABA restarted 2019	G3
14	Male	no	no	no	4.29l/84%	3.10l/73%	1	ICS, LABA, montelukast, tiotropium, salbutamol weekly, nasal steroid	G4
15	Female	no	no	no	3.52l/83%	2.92l/83%	4	ICS, LABA, tiotropium	G4
16	Female	no	no	no	3.60l/96%	2.90l/96%	2	ICS, LABA	G3
17	Male	no	N.A.	yes	5.27l/91%	3.45l/74%	2	ICS, salbutamol weekly	G2
18	Male	yes	no	no	5.60l/96%	4.50l/101%	4	ICS, salbutamol during exercise	G2
19	Female	no	no	no	2.76l/89%	2.19l/88%	2	ICS, LABA, salbutamol during exercise	G3
20	Male	no	yes	yes	5.44l/96%	4.23l/92%	4	ICS, montelukast, antihistamine, nasal steroid,	G2
21	Female	no	no	no	3.05l/93%	2.13l/81%	4	ICS, LABA	G3

1: Forced expiratory volume in one second (FEV1)/Forced vital capacity (FVC) + 12% or more with salbutamol (increase in FEV_1_ > 12% with salbutamol); 2: Peak expiratory flow (PEF) + 15%/60 l or more; minimum 3 times with salbutamol; 3: PEF + 20%/60% or more; minimum 3 times day variation, excessive variability in twice-daily PEF over 2 weeks (>20%); 4: histamine provocation test positive. Inhaled corticosteroid (ICS). Long-acting β_2_-agonist (LABA).

**Table 2:** Toxicity data for dust and cultured dust samples from the work spaces of 21 patients who acquired occupational asthma during a renovation process.

Patient Number	Dust Toxicity	Fungal Toxicity of Cultured Dust	Bacterial Toxicity of Cultured Dust
1	S (10)	NT (61)	S (5)
2	VS (6)	S (1)	S (5)
3	S (10)	N.A.	S (1)
4	VS (6)	S (1)	S (5)
5	S (9)	M (19)	S (9)
6	VS (6)	S (1)	S (5)
7	VS (6)	S (1)	S (5)
8	S (9)	NT (42)	S (1)
9	S (9)	NT (42)	S (1)
10	NT (19)	M (36)	S (5)
11	M (13)	S (14)	S (5)
12	M (16)	S (12)	S (5)
13	N.A.	NT (38.0)	S (3)
14	S (9)	S (1)	S (5)
15	S (10)	N.A.	S (1)
16	N.A.	NT (42)	S (2)
17	S (9)	NT (42)	S (1)
18	S (9)	NT (42)	S (1)
19	S (10)	NT (54)	S (2)
20	M (17)	M (37)	S (7)
21	NT (73)	S (9)	M (17)

The criteria for toxicity are based on previous research [13,14]. The categories of degrees of toxicity (VS, S, M, NT) are based on the customer database by dividing the material into quartiles (Inspector Sec Ltd. Research Services). Dust toxicity: VS = very strong toxicity (EC_50_ ≤ 6 µg/mL); S = strong toxicity (EC_50_ = 6.5–12.5 µg/mL); M = mild toxicity (EC_50_ 13–18 µg/mL); NT = not toxic (EC_50_ > 18 µg/mL). Fungal toxicity of cultured dust: S = strong toxicity (EC_50_ ≤ 16 µg/mL); M = mild toxicity (EC_50_ 17–37 µg/mL); NT = not toxic (EC_50_ > 37 µg/mL). Bacterial toxicity of cultured dust: S = strong toxicity (EC_50_ ≤ 9 µg/mL); M= mild toxicity (EC_50_ 10–19 µg/mL); NT = not toxic (EC_50_ > 19 µg/mL). Numbers in parenthesis: precise calculated values, µg/mL. N.A. = not available.
